# Role of *Enterococcus faecalis* in refractory apical periodontitis: from pathogenicity to host cell response

**DOI:** 10.1080/20002297.2023.2184924

**Published:** 2023-03-01

**Authors:** Zilong Deng, Binbin Lin, Fan Liu, Wanghong Zhao

**Affiliations:** a Department of Stomatology, Nanfang Hospital, Southern Medical University, Guangzhou, China; b School of Stomatology, Southern Medical University, Guangzhou, China

**Keywords:** *Enterococcus faecalis*, macrophage, osteoblast, refractory apical periodontitis, virulence, regulated cell death, differentiation, polarisation

## Abstract

Background: Refractory apical periodontitis (RAP) is an oral infectious disease characterised by persistent inflammation, progressive alveolar bone destruction, and delayed bone healing. RAP has received increasing attention, because it cannot be cured after repeated root canal therapies. The aetiology of RAP is related to the complex interplay between the pathogen and its host. However, the exact pathogenesis of RAP remains unclarified and includes several factors, such as microorganism immunogenicity, host immunity and inflammation, and tissue destruction and repair. Enterococcus faecalis is the dominant pathogen involved in RAP, and has evolved multiple strategies to ensure survival, which cause persistent intraradicular and extraradicular infections.

Objective: To review the crucial role of E. faecalis in the pathogenesis of RAP, and open new avenues for prevention and treatment of RAP.

Methods: The PubMed and Web of Science databases were searched for pertinent publications, employing the search terms “Enterococcus faecalis”, “refractory apical periodontitis”, “persistent periapical periodontitis”, “pathogenicity”, “virulence”, “biofilm formation”, “dentine tubule”, “immune cell”, “macrophage”, and “osteoblast”.

Results and Conclusion: Besides its high pathogenicity due to various virulence mechanisms, E. faecalis modulates the macrophage and osteoblast responses, including regulated cell death, cell polarisation, cell differentiation, and inflammatory response. An in-depth understanding of the multifaceted host cell responses modulated by E. faecalis will help to design potential future therapeutic strategies and overcome the challenges of sustained infection and delayed tissue healing in RAP.

## Introduction

Apical periodontitis (AP) is an inflammatory disease characterised by periapical tissue injury of the affected teeth caused by microbial infection, which often requires root canal therapy (RCT) to control infection and eliminate inflammation. A meta-analysis investigating the global prevalence of AP showed that half of the adult population worldwide has AP, while the frequency of AP in root-filled teeth is as high as 39% [1]. Failure to heal the lesion after RCT prompts patients to seek further treatment. Refractory apical periodontitis (RAP) is chronic AP that persists after repeated routine RCT. Although multiple biological factors, such as intraradicular and extraradicular microbial infections and exogenous root canal filling materials, can lead to persistence of periapical lesions after RCT [2], the most common aetiology of RAP is uncontrolled pathogen infection, posing a challenge to clinical therapy [3,4]. Accumulating evidence has shown that the complex interactions between pathogenic microorganisms and the host are important in the pathogenesis of RAP. Therefore, a more comprehensive understanding of the specific mechanisms of pathogen-modulated host cell responses is required to determine potential therapeutic targets to control infection and inflammation, promote tissue repair, and provide new insights into prevention and treatment of RAP.

*Enterococcus faecalis*, a gram-positive facultative anaerobic bacterium, is an opportunistic pathogen that is commonly found in the human oral cavity and gastrointestinal tract. Evidence has shown that *E. faecalis* is one of the most common pathogenic microorganisms in root canals with RAP, as it is frequently isolated from root canals with endodontic failure [5–10]. *E. faecalis* survives for a long time in root canals, because it can tolerate a highly alkaline and oligotrophic environment to form biofilms [11,12], invade deep into the dentin tubules [13], and easily evade phagocytosis by host cells [14]. Moreover, *E. faecalis* can also be detected in the periradicular lesions and extraradicular biofilms in patients with RAP [15]. Lipoteichoic acid (LTA), a cell wall component of gram-positive bacteria that acts as an important virulence factor, can trigger cascades resulting in pro-inflammatory cytokine release and periapical tissue damage by binding to targets, either specifically, to CD14 and to Toll-like receptors (TLRs), or non-specifically, to membrane phospholipids [16]. *E. faecalis* LTA participates in biofilm formation and adhesion to eukaryotic cells due to its alanylation that modulates the net surface charge of the bacteria [17]. Significant statistical correlations were found between levels of LTA and clinical features (periapical lesion areas and symptoms) [4,6]. Collectively, the contribution of *E. faecalis* and its virulence factor, LTA, in the pathogenesis of RAP is pivotal.

Macrophages function as crucial responsive cells in the host’s innate immune system. They recognise and phagocytose pathogens, present antigens, secrete a variety of cytokines or chemokines to regulate the body’s immune and inflammatory responses, and polarise or differentiate into different subtypes of cells to regulate tissue homeostasis [18]. Infection by diverse pathogens induces secretion of inflammatory cytokines and chemokines by tissue-resident macrophages and recruitment of the circulating monocytes from the blood to the sites of infection where they differentiate into macrophages to eliminate the microbes [19]. Studies have supported that macrophage migration, activation and mitophagy play a vital role in the progression of RAP [20–22]. The symptomatic or asymptomatic forms of AP, as well as the development of AP towards apical granulomas or radicular cysts, may be related to macrophage polarisation [23,24]. Osteoblasts are essential in all stages during bone remodelling; maintaining the number and activity of osteoblasts have a significant impact on the repair efficiency in periapical bone loss [25]. However, RAP-related bacteria and their virulence factors affect the fate and function of osteoblasts [26,27]. To date, studies have uncovered several links between *E. faecalis* and these two cell types. Integrating and summarising the evidence can improve our understanding of the pathogenic mechanisms of *E. faecalis* in RAP and further reveal the complex causes of persistent periapical inflammation and delayed bone healing, thus helping to optimise clinical treatment strategies and improve the success rate of treatment of periapical diseases.

In this review, we summarise the pathogenic properties of *E. faecalis* and its regulatory mechanisms in host macrophages and osteoblasts. We focused on the underlying mechanisms of *E. faecalis*-modulated responses in these two cell types, including regulated cell death (RCD), cell differentiation, cell polarisation, and inflammatory response, and their roles in the progression of RAP.

## Pathogenic properties of *E. faecalis* associated with RAP

RAP is due, in part, to the long-term survival of *E. faecalis* in the treated complex root canal system. Accumulating evidence has indicated different causative factors associated with *E. faecalis* pathogenicity. Since biofilm formation facilitates survival and virulence of microorganisms, herein, we discuss the ability of *E. faecalis* to invade dentinal tubules and form biofilms in adverse environments after RCT.

### Biofilm formation under unfavourable conditions

- *E. faecalis* contains many virulence factors, such as cytolysin activator (cylA), gelatinase (gelE), extracellular surface protein (esp), aggregation substance (asa), adhesion of collagen (ace), *E. faecalis* regulator B (fsrB), and endocarditis antigen A (efaA), which are involved in bacterial adhesion, biofilm formation, resistance to killing, and tissue damage [28–30]. The genes encoding these virulence factors have been detected in some root canal isolates [28,31–33]. Among them, fsrB, asa, ace, efaA, esp, and gelE are significantly linked to adhesion and biofilm formation [34]. Under starvation or alkaline conditions, the transcript levels of certain biofilm formation-related virulence genes were upregulated, which may account for the resistance of *E. faecalis* to alkaline root canal medications [35,36].
- *E. faecalis* maintains its biofilm-forming capacity in many ways under stress. In starvation and alkaline environments, the cell-surface hydrophobicity of *E. faecalis* increased, which is conducive to bacteria adhesion and aggregation [35,36]. Under such conditions, changes occurred in the synthesis of water-soluble exopolysaccharides, water-insoluble exopolysaccharides, and intracellular polysaccharides, thus contributing to *E. faecalis* defence against environmental stress [35–37]. Moreover, cells grown in biofilms during starvation are characterised by increased protein synthesis and decreased nucleic acid levels [38]. After alkaline stress, the bacterial cell wall structure is altered, potentially making bacteria less susceptible to recognition and phagocytosis by macrophages [14]. Furthermore, the multispecies community facilitates the starvation resistance of *E. faecalis* and biofilm formation in the root canals [39]. According to Chavez *et al*., *E. faecalis* strain that produced higher levels of gelE and three serine proteases outcompeted *Lactobacillus salivarius* and *Streptococcus gordonii* in multispecies biofilms [40]. Restriction of iron availability is a defence strategy by the host at infection sites. However, Tan *et al*. found that *E. faecalis* can lower the environmental pH by exporting lactic acid to antagonize *P. aeruginosa* growth within biofilms under iron-restricted conditions [41].

In addition to facilitating survival in root canals or periapical tissues, biofilm growth of *E. faecalis* leads to low-grade chronic inflammation, which may be associated with and RAP. Mathew *et al*. found that biofilm-derived *E. faecalis* cells exhibited higher intracellular survival potential and produced lower levels of the pro-inflammatory mediators IL-6 and TNF-α, compared with planktonic cells [42]. Similarly, macrophages exposed to monospecies biofilm matrix components, namely extracellular DNA and extracellular polysaccharides, released less TNF-α, IL-6, and NO than those exposed to planktonic bacteria and lipopolysaccharides [43].

In general, a variety of factors influence biofilm formation by *E. faecalis* under harsh root canal conditions, including expression of various virulence genes, alterations in intracellular and extracellular biochemical compositions, and interactions between microorganisms. These findings will inform further research on how to control infections by inhibiting bacterial biofilm formation. Biofilm inhibitors will be the focus of research into RAP preventative approaches. Novel targeted molecule inhibitors have been investigated for endodontic treatment. For example, antisense *walR* RNA, which interferes with the expression of the two-component signal transduction systems of *E. faecalis*, reduces virulence gene expression, exopolysaccharide synthesis, and biofilm formation [44,45]. Furthermore, nanomaterial-carried antisense *walR* RNA increases bacterial susceptibility to root canal disinfectants and inhibits the pathogenicity of *E. faecalis* in RAP [46,47].

### Dentinal tubule invasion and persistence within the host

Another important pathogenic property of *E. faecalis* is the ability to invade dentinal tubules, making it difficult to completely remove the pathogen, thus leading to reinfection [13]. Researchers observed that devitalised *E. faecalis* cells were still able to migrate into dentinal tubules, suggesting that invasion of dentinal tubules by *E. faecalis* may involve an electrokinetic and osmotic process [48,49]. Under alkaline and glucose starvation stress conditions, *E. faecalis* is able to form biofilms but has a significantly decreased invasive ability to dentinal tubules, which may be associated with enhanced cell-surface hydrophobicity [50].

It has been reported that root canal irrigation with different protocols seemed unable to completely eradicate *E. faecalis* bacteria residing in dentinal tubules [51,52]. Residual *E. faecalis* at the time of root filling can lead to subsequent reinfection [53]. The persistence of *E. faecalis* is thought to result from its ability to enter the viable but nonculturable state (the VBNC state, being incapable of cellular division but stay live) when exposed to stressful environment after root canal disinfection, thus becoming insensitive to disinfectants, waiting for the opportunity to restore growth and proliferation, and then causing reinfection [54–56]. Deeper understanding of the VBNC state should result in novel management for RAP. The identification of the molecular determinants required for the transition of *E. faecalis* to the VBNC state may direct development of therapeutics that target the chronicity of infections by this bacterium.

## *E. Faecalis*-modulated host cell responses

Periapical lesions can be regarded as the result of the fight and interactions between pathogenic insults and the local defence system of the periapical tissue. In RAP, pathogenic microorganisms and their toxins inside and outside the root canals continuously attack the periapical tissue, perpetuate local lesions, and delay healing. As research has progressed, investigators have recently focused on the role of host cell responses modulated by *E. faecalis* infection in the pathogenesis of RAP and related mechanisms.

### *E. faecalis* modulates immune responses of macrophages

Studies on the mechanisms of macrophage immune responses modulated by *E. faecalis* in RAP have focused on three aspects, namely, regulated cell death (RCD), macrophage polarisation, and macrophage differentiation, all of which involve inflammatory response (Table 1). *E. faecalis* LTA partially contributes to the inflammatory response. It can be recognised by TLR2 on the surface of macrophages and induce NLRP3 inflammasome activation mainly through the nuclear factor kappa B (NF-κB) followed by cytokine secretion [66,67].

**Table 1.** Summary of *Enterococcus faecalis*-modulated macrophage responses.

Host cell responses	Stimuli	Cells	Upregulate	Downregulate	Effect	Reference
Regulated cell death	*E. faecalis* (ATCC 33,186)	THP-1 macrophages	NLRP3/CASP-1 activation, GSDMD cleavage	/	inducing pyroptosis	[57]
	*E. faecalis* (CA1, CA2 and OG1RF)	Raw264.7	CASP-3, NLRP3/CASP-1, and RIPK3/MLKL activation; GSDMD cleavage	/	inducing apoptosis, pyroptosis, and necroptosis	[58]
	*E. faecalis* (E99, MMH594 and OG1RF)	Raw264.7	NO and ROS production	conversion of LC3-I to LC3-II	resisting to autophagy	[59]
	*E. faecalis* (E99, MMH594, V583 and OG1RF)	Raw264.7	PI3K/Akt activation, Bcl-2 expression	CASP-3 activation, Bax expression	inhibiting apoptosis	[60]
	*E. faecalis* (ATCC 29,212)	THP-1 macrophages	NLRP3/CASP-1 activation, GSDMD cleavage	/	inducing pyroptosis	[61]
	LTA from *E. faecalis* (strain used unknown)	Raw264.7	Beclin 1 and LC3-II expression	PI3K/Akt/mTOR activation	inducing autophagy	[62]
Polarisation	*E. faecalis* (OG1RF and OG1RF derivative)	Raw264.7	/	carbohydrate metabolism	inhibiting polarisation to M1 phenotype	[63]
	*E. faecalis* (ATCC 29,212)	BMSCs-derived macrophages	IL-10 secretion	IL-1β and IL-12 secretion	inducing an atypical M1-like phenotype	[64]
	*E. faecalis* (ATCC V583)	THP-1 macrophages	internal ROS	external ROS	inducing polarisation to M2 phenotype	[65]
Inflammatory response	LTA from *E. faecalis* (strain used unknown)	Raw264.7	NF-κB and NLRP3 activation	/	contributing to the inflammatory response	[66]
	LTA from *E. faecalis* (ATCC 29,212)	Raw264.7	NF-κB activation, TNF-α secretion	/	contributing to the inflammatory response	[67]
Differentiation of macrophages into osteoclasts	*E. faecalis* (OG1RF) or HKEF (OG1RF)	Raw264.7	/	*Acp5*, *Ctsk*, *c-fos*, *Dcstamp* and *Atp6v0d2* gene expression	inhibiting osteoclast differentiation	[68]
	HKEF (ATCC 29,212)	MC3T3-E1/Raw264.7 co-culture system	ephrinB2-EphB4 bidirectional signalling, *NFATc1* gene expression	/	promoting osteoclast differentiation	[69]
	HKEF (ATCC 29,212)	Raw264.7	p38 and ERK1/2 activation	/	promoting osteoclast differentiation	[70]
	HKEF (ATCC 29,212)	BMMs	/	c-Fos and NFATc1 expression	inhibiting osteoclast differentiation	[71]
	LTA from *E. faecalis* (ATCC 29,212)	Raw264.7	JAK2/STAT3 activation	/	promoting osteoclast differentiation	[72]
	three LTAs from *E. faecalis* (P25RC, P52Sa and ATCC 29,212)	BMMs	*NFATc1* and *RBP-J* gene expression	*c-Fos* and *NFATc1* gene expression	inhibiting osteoclast differentiation	[73]
	LTA from *E. faecalis* (ATCC 29,212)	BMMs	/	c-Fos and NFATc1 expression	inhibiting osteoclast differentiation	[74]

HKEF, heat-killed *E. faecalis*; BMSCs, bone marrow stem cells; BMMs, bone marrow-derived macrophages; CA1, CA2, and P25RC, three root canal isolated strains; V583, the first clinical isolate with vancomycin resistance; E99, a clinical isolate from a urinary tract infection; MMH594, a clinical isolate that caused multiple infections; OG1RF and P52Sa, two oral isolated strains.

#### *E. faecalis* and RCD in macrophages

**Figure 1.**
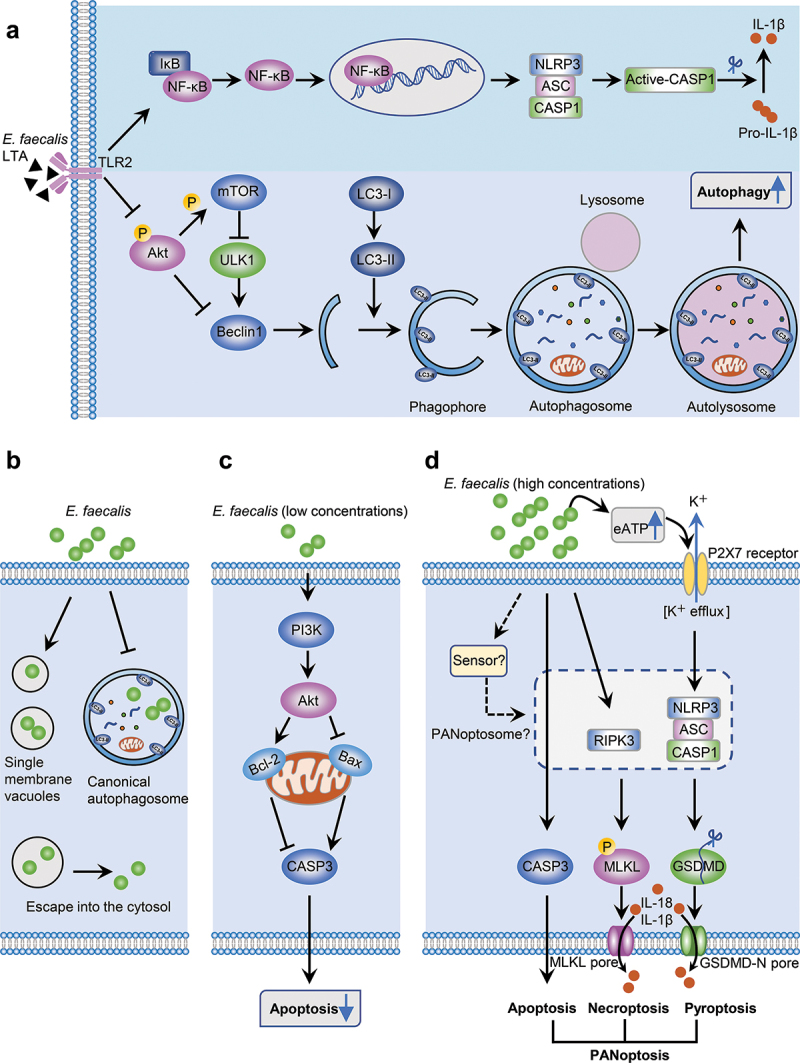
Schematic diagram of *E. faecalis*-modulated regulated cell death and inflammatory response in macrophages. a: The virulence factor, *E. faecalis* lipoteichoic acid, contributes to pro-inflammatory responses via modulating NLRP3 inflammasome activation by the NF-κB pathway. *E. faecalis* lipoteichoic acids also promotes autophagy in macrophages, weakening the killing effect of macrophages. b: Most engulfed *E. faecalis* bacteria are enclosed by single membrane vesicles without being transported into classic double-membraned autophagosomes in macrophages, although the molecular mechanisms remain unclear. c: *E. faecalis* inhibits macrophage apoptosis at low bacterial concentrations, thus prolonging bacteria survival. d: High concentrations of *E. faecalis* may induce macrophage PANoptosis and promote inflammation. However, the specific sensor that induces the assembly of the PANoptosome and the complete components that interact to form the PANoptosome are unknown.

Phagocytosis and RCD are required for pathogen elimination. As macrophages are important phagocytes, *E. faecalis*-modulated RCD in macrophages is an important mechanism involved in RAP. The Nomenclature Committee on Cell Death updated the cell death classification in 2018, defining more than 10 RCDs from a molecular mechanism perspective [75]. In the absence of any exogenous environmental perturbation, RCD operates as a physiological program for the organism’s development or maintenance of tissue homeostasis. On the other hand, RCD can be initiated upon exposure of cells to endogenous or exogenous stimuli, playing a role in the elimination of microbes-infected or damaged cells through a specific molecular mechanism. However, excessive immune responses caused by regulated lytic cell death can lead to tissue injury [75]. Regarding the pathogenic mechanisms of macrophage RCD modulated by *E. faecalis* infection, current studies have suggested that *E. faecalis* infection can modulate macrophage apoptosis, pyroptosis, necroptosis and autophagy-dependent cell death through different signalling pathways (Figure 1).

Apoptosis is a highly conserved process that includes two pathways, intrinsic and extrinsic, aimed at maintaining tissue homeostasis by eliminating senescent and damaged cells [76]. Caspase-3 (CASP-3) is activated in both intrinsic and extrinsic apoptosis and activated CASP-3 can cleave more than 500 cellular substrates to execute the apoptotic program [77]. In contrast, an important function of activated phosphatidylinositol-3-kinase (PI3K) in cells is to inhibit apoptosis, and protein kinase B (Akt) is an anti-apoptotic factor that mediates PI3K-dependent cell responses [78]. A previous study indicated that four different *E. faecalis* strains reduced macrophage apoptosis by inhibiting CASP-3 activation, at a multiplicity of infection (MOI, the ratio of bacteria to host cells) of 10:1 [60]. This anti-apoptotic process is dependent on the activation of the PI3K/Akt signalling pathway which leads to increased expression of the anti-apoptotic factor Bcl-2 and reduced expression of the pro-apoptotic factor Bax. Similarly, another study showed that *E. faecalis* infection of bone marrow-derived stem cells prior to their differentiation into macrophages, with an MOI of 1:1 for a prolonged time to mimic chronic infection, decreased apoptotic activity in subsequently differentiated macrophages [64]. In addition, researchers found no macrophage death in the early stages of infection through real-time cell death analysis; however, when *E. faecalis* proliferation reached a high concentration (real-time MOI>10^3^), CASP-3 transcript and protein expression were significantly upregulated, and macrophage apoptosis was induced [58]. These results indicate that at low bacterial concentrations, *E. faecalis* may inhibit apoptosis in macrophages to survive for an extended time and promote infection spread. But the relationship between *E. faecalis* infection and macrophage apoptosis is so complex that further studies are warranted.

Unlike apoptosis, pyroptosis and necroptosis are two pro-inflammatory necrotic RCDs whose key morphological feature is cell membrane rupture [77]. In terms of pyroptosis, damage-related molecular patterns (DAMPs) or bacterial endotoxins activate inflammasomes in phagocytes and then activate inflammatory caspases (CASP-1/-4/-5 in humans; CASP-1/-11 in mice) to cleave gasdermin-D (GSDMD), which generates pores in the plasma membrane and leads to the release of mature IL-1β, IL-18, and DAMPs [79–81]. Although different inflammasomes and inflammatory caspases are associated with pyroptosis, current studies have only confirmed that *E. faecalis* induces CASP-1- and NLRP3 inflammasome-dependent macrophage pyroptosis [57,58,61]. With regard to necroptosis, tumour necrosis factor (TNF) binds to TNF receptor 1 in bacteria-infected cells, followed by receptor-interacting protein kinase (RIPKs) activation. When CASP-8 is compromised by z-VAD-fmk, RIPK1 and RIPK3 are activated to form necrosomes, which phosphorylate mixed lineage kinase domain-like protein (MLKL) and disrupt the plasma membrane, resulting in the release of DAMPs [82]. Notably, growing evidence has highlighted crosstalk among apoptosis, pyroptosis, and necroptosis. Recently, these observations have led to the emergence of the concept of PANoptosis, defined as a lytic inflammatory RCD pathway driven by the PANoptosome [83–85]. A study showed that when *E. faecalis* root canal isolates (CA1 and CA2) and OG1RF strain proliferated to high concentrations, they activated apoptosis, pyroptosis, and necroptosis in macrophages to various degrees, possibly associated with PANoptosis [58]. Most of the virulence factors in CA2 strains are related to capsule formation, which may enhance their immune clearance resistance, as macrophages infected with CA2 began to experience cell death later than those in the other two infection groups in that study. Additionally, expression of PANoptosis-related genes was lower in CA2-infected macrophages than in OG1RF-infected macrophages. However, real-time cell death analysis indicated that the CA2 and OG1RF infection groups had similar final macrophage cell death rates, suggesting that PANoptosis in *E. faecalis*-infected macrophages may target immune-escaping pathogens [58]. Hence, PANoptosis has far-reaching implications for infection control. Immune escape mechanisms allow *E. faecalis* to sustain persistent infections, and activation of PANoptosis may help to more effectively prevent pathogens from evading immune clearance. However, our understanding of *E. faecalis*-induced PANoptosis is limited. Further studies should determine the specific sensor that recognises *E. faecalis* and initiates assembly of the PANoptosome. The PANoptosome components and their interaction network are still unclear. Furthermore, an important remaining question is whether and how we can reduce the release of excessive pro-inflammatory cytokines when controlling infections via modulation of PANoptosis.

Autophagy, as a fundamental cellular degradation mechanism in eukaryotes, is equally important in innate immune defence and pathogenic microbe clearance [86,87]. Bacteria induce autophagy by stimulating innate immune receptors such as TLRs. When bacteria are phagocytosed in intact vacuoles, autophagosomes mature to autolysosomes through an autophagic process termed LC3-associated phagocytosis [86]. Autophagy is involved in the pathogenesis of AP and is partially associated with apoptosis [88,89]. LTA from *E. faecalis* can activate macrophage autophagy through Beclin1 and inhibition of the PI3K/Akt/mTOR pathway, which attenuates macrophage-mediated killing [62]. However, classical double-membrane autophagosomes were not observed in *E. faecalis*-infected macrophages, and some bacteria escaped from the monolayer membrane vesicles [58,59]. Enhancing autophagy can inhibit the intracellular survival of *E. faecalis* in macrophages and may be a potential new therapy for the treatment of RAP. However, the specific immune mechanisms and key molecules involved in *E. faecalis*-modulated autophagy should be identified.

#### *E. faecalis* and polarisation of macrophages

M1 and M2 macrophages have opposite polarities and the ability to mediate killing and repair responses, respectively. They coexist in various tissues throughout the body to regulate immune responses. Macrophages produce the killing molecule nitric oxide (NO) and the repair-promoting molecule ornithine by metabolising arginine to initiate M1- and M2-type responses, respectively [90]. M1 macrophages activate innate and adaptive immunity, whereas M2 macrophages regulate tissue regeneration and participate in the clearance of apoptotic vesicles and immunosuppression [90]. The clinical impact of M1 killing and M2 repair responses is enormous, and controlling the balance between M1 and M2 macrophages may be of great therapeutic benefit in cancer, infection, and chronic inflammation [91,92].

Of note, metabolism plays a key role in macrophage activation and polarisation [93,94]. A recent study reported the metabolic interaction between *E. faecalis* and macrophages utilizing transposon insertion sequencing (TIS) coupled with transcriptome sequencing. TIS was performed using a transposon insertion mutant library of *E. faecalis* OG1RF, to comprehensively assess metabolism-related genetic determinants of *E. faecalis* resistance to macrophage-mediated killing [63]. Analysis of the sequencing results showed that a quantity of metabolism-related mutants in the library, especially those associated with mannose and fructose metabolism, exhibited survival enhancement in RAW264.7 cells. The authors’ further exploration validated that *E. faecalis* downregulates carbohydrate metabolism to promote its survival in macrophages. Mechanistically, attenuation of carbohydrate metabolism in *E. faecalis* decreases the polarisation of macrophages to M1 phenotype to reduce NO production, thus facilitating the resistance of *E. faecalis* to macrophage-mediated killing [63].

Studies have suggested that M1 macrophages induce production of pro-inflammatory cytokines, such as IL-1β, IL-6, IL-12, and TNF-α, while IL-4, IL-10, and IL-13 polarise macrophages toward the M2 phenotype [91]. Intriguingly, *E. faecalis* induces macrophage polarisation into an atypical M1-like phenotype, which expresses an aberrant cytokine mRNA profile with reduced levels of the pro-inflammatory cytokines IL-1β and IL-12 and increased levels of the immunosuppressive cytokine IL-10. This atypical M1-like phenotype was retained even in the presence of IL-4 and IL-13 [64]. Furthermore, macrophages that remain viable and functional after *E. faecalis* infection undergo M2 polarisation [65]. Macrophages usually exhibit plasticity between M1 and M2 subsets, where the two phenotypes can be interconverted in response to different stimuli. The M1 to M2 phenotypic shift is required for inflammation resolution and healing [91]. Atypical M1-phenotype, highly expressing CD38 and protein IRF5 (specific markers for M1 polarisation) but expressing an M2-like cytokine profile, may represent an intermediate state within the macrophage phenotypic spectrum, which is suggestive of the inhibited plasticity and the altered function of macrophages modulated by *E. faecalis* infection. These studies provide new insights into the mechanisms involved in intracellular survival of *E. faecalis*.

In addition, heat-killed *E. faecalis* (HKEF) stimulation downregulated IL-12 and upregulated IL-10, TNF-α, and NO production in the presence of recombinant interferon-γ, without affecting MCP-1, IL-1α, and IL-6 production [95]. Since HKEF reproduces part of virulence factors of living *E. faecalis*, increased expression of TNF-α and NO suggested that *E. faecalis* virulence factors may contribute to M1 polarisation, triggering pro-inflammatory and antimicrobial responses during the initial stages of infection. Downregulation of IL-12 and upregulation of IL-10 are consistent with the findings by Mohamed *et al* [64]. And may be related to virulence mechanisms of diminished immune response and macrophage polarisation to the M2 or atypical M1 phenotype in the late response to infection with *E. faecalis*.

**Figure 2.**
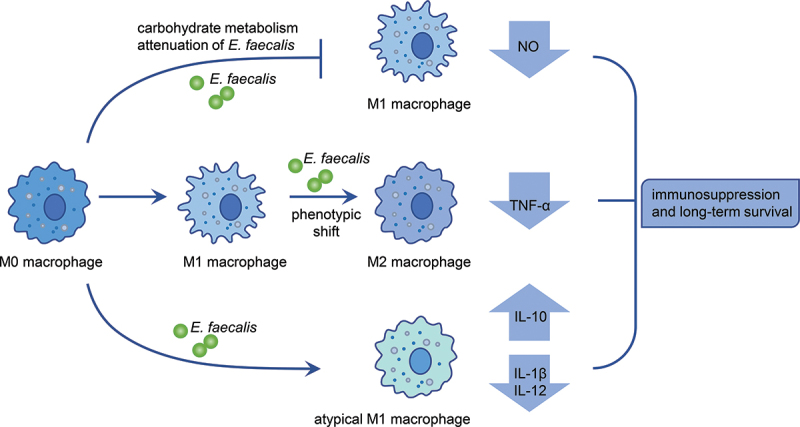
Schematic diagram of *E. faecalis*-modulated macrophage polarisation showing immune evasion strategies. *E. faecalis* shifts macrophage polarisation towards an M2-like phenotype or atypical M1-like phenotype with an altered cytokine profile. During infection, *E. faecalis* reduces carbohydrate metabolism, thus attenuating M1 macrophage-mediated killing.

In summary, it has been preliminarily shown that *E. faecalis* affects macrophage polarisation (Figure 2), thus evading immune clearance and leading to persistent bacterial infection and chronic RAP. The regulatory factors and precise mechanisms should be further investigated, because controlling the balance between M1 and M2 macrophages may be beneficial for RAP treatment.

#### *E. faecalis* and osteoclast differentiation of macrophages

RAP is essentially a disease of inflammatory bone destruction, which is widely considered as an imbalance of the bone remodelling process regulated by osteoclasts and osteoblasts. Macrophages can differentiate into osteoclasts as osteoclast precursor cells, affecting bone healing in periapical tissues [96]. Osteoclast activation and differentiation are regulated by three members of the TNF ligand and receptor superfamily: osteoprotegerin (OPG), receptor activator of nuclear factor-κB ligand (RANKL), and RANK [97]. When RANKL binds to its receptor RANK on the surface of osteoclast precursor cells, it promotes osteoclast formation, whereas OPG binds to RANKL and blocks the RANKL-RANK interaction. The ratio of RANKL to OPG influences the osteoclast differentiation status and bone mass [98]. *E. faecalis* may promote RANKL-dependent osteoclast formation and Sema4D protein expression via the p38 and ERK1/2 MAPK signalling pathways [70]. *E. faecalis*-derived LTA possibly promotes differentiation of RAW264.7 macrophages into osteoclasts, in association with the Janus kinase 2/signal transducer and activator of transcription 3 (JAK2-STAT3) signalling pathway [72]. In a macrophage/pre-osteoblast co-culture system, HKEF may induce osteoclast differentiation through the ephrin ligand B2-Eph receptor B4 (ephrinB2-EphB4) bidirectional signalling [69]. Conversely, there are different conclusions reported. Some researchers have found that *E. faecalis* may attenuate the ability of macrophages to differentiate into osteoclasts, thus maintaining their ability to phagocytose pathogens and induce inflammation [68,71]. LTA from *E. faecalis* may inhibit RANKL-induced osteoclast formation via the transcription factor RBP-J [73,74].

Whether *E. faecalis* exerts a positive or negative regulatory effect on osteoclastogenesis of macrophages is unclear, according to current studies. These discrepant results may be attributed to the fact that no other immune cells are tested in *in-vitro* experiments, which cannot mimic the complex environment of the human oral cavity. Immunomodulators, such as IL-6, IL-1, and TNF-α, play a crucial role in the regulation of osteoclastogenesis and bone resorption, and dysregulated or prolonged immune responses often affect bone metabolism [99]. Based on this knowledge, scholars supposed that bone destruction caused by *E. faecalis* infection is mediated mainly by host cellular immune responses [68]. It is plausible that *E. faecalis* does not directly promote macrophage differentiation into osteoclasts, but rather that secreted cytokines mediate the bone destruction process. Thus, there is a need to develop cell co-culture models that better mimic the local microenvironment of human periapical tissues, and more advanced assay techniques to explore the underlying mechanisms.

### *E. faecalis* modulates cell death and differentiation of osteoblasts

Osteoblasts, which are crucial for bone matrix protein production and bone mineralisation, can differentiate into osteocytes, the most abundant cells in the bone [100,101]. Osteoblast number and activity are essential for the recovery of periapical bone defects. Recently, the effect of *E. faecalis* on osteoblast RCD and osteoblastic differentiation has also attracted the attention of researchers (Table 2).

**Table 2.** Summary of *Enterococcus faecalis*-modulated osteoblast responses.

Host cell responses	Stimuli	Cells	Upregulate	Downregulate	Effect	Reference
Regulated cell death	*E. faecalis* (OG1RF, ATCC 47,077)	MG63	CASP-3/-8/-9 activation, survivin expression	death receptor 6 expression	inducing intrinsic and extrinsic apoptosis	[102]
	*E. faecalis* (ATCC 29,212)	MG63	RIPK3/MLKL activation	/	inducing necroptosis	[103]
	*E. faecalis* (ATCC 33,186)	MG63	NLRP3/CASP-1 activation	/	inducing apoptosis and pyroptosis	[104]
	*E. faecalis* (OG1RF, CE, and CA)	MC3T3-E1	CASP-3 activation, Bax expression	Bcl-2 expression	inducing apoptosis	[105]
	LTA from *E. faecalis* (strain used unknown)	MG63	CASP-3 activation, Bax expression	Bcl-2 expression	inducing apoptosis	[106]
Osteoblastdifferentiation	HKEF (ATCC 29,212, P25RC)	MC3T3-E1	p38 and ERK1/2 activation	*Runx2*, *ALP*, *OCN* and *COL1* gene expression	inhibiting osteoblast differentiation	[107]
	HKEF (ATCC 29,212)	osteoblast precursors from mouse calvaria	chemokine KC and MCP-1 secretion	*Runx2*, *osterix*, *β-catenin*, *OCN*, and *COL1* gene expression	inhibiting osteoblast differentiation	[108]
	*E. faecalis* (FRs 112)	ovine osteoblast-like cells	/	ALP activity, calcium deposition, *OCN* gene expression	inhibiting osteoblast differentiation	[109]

HKEF, heat-killed *E. faecalis*; OG1RF, an oral isolated strain; CE, CA, and FRs 112, three root canal isolated strains.

#### *E. faecalis* and RCD in osteoblasts

Studies on RCD in osteoblasts have indicated that *E. faecalis* may induce osteoblast PANoptosis (apoptosis, pyroptosis, and necroptosis) via different signalling pathways.

To explore how *E. faecalis* affects osteoblast apoptosis, researchers initially focused on the virulence factor LTA and found that it inhibits proliferation of human osteoblast-like MG63 cells and induces apoptosis [106]. Later, other investigators found that clinically isolated *E. faecalis* induces apoptosis in MC3T3 osteoblasts by upregulating expression of Bax and CASP-3, and downregulating expression of Bcl-2 [105]. Further study suggested that *E. faecalis* induces and accelerates osteoblast apoptosis in an MOI-dependent and time-dependent manner, activating not only the CASP-8-dependent extrinsic apoptotic pathway, but also the CASP-9-dependent intrinsic apoptotic pathway. Specifically, the intrinsic pathway is time-dependent, whereas the extrinsic pathway is more sensitive to the MOI [102]. In addition, *E. faecalis* promoted osteoblast pyroptosis and apoptosis via the NLRP3 inflammasome, which was inhibited after downregulation of the *NLRP3* gene with small interfering RNAs [104]. However, significant activation of CASP-1 and GSDMD, key molecules in the pyroptotic pathway, was not observed in a study by Li *et al*., indicating that *E. faecalis* may not induce pyroptosis. The results of these two studies are contradictory and require further investigation. New evidence showed that *E. faecalis* infection induces necroptosis in MG63 cells via the RIPK3/MLKL signalling pathway. Silencing MLKL using inhibitors or short hairpin RNA significantly reduced osteoblast death [103]. Taken together, *E. faecalis* may induce PANoptosis in osteoblasts.

Recently, a polymerase chain reaction (PCR) array analysis of 84 apoptosis-related genes in *E. faecalis*-infected human calvarial osteoblasts revealed that Bcl-2 family members acted as regulators of osteoblast apoptosis. Therefore, Bcl-2 family members may be potential therapeutic targets for RAP [110]. Notably, utilising inhibitors targeting pyroptosis or necroptosis-related molecules may have a significant impact on the resolution of *E. faecalis*-induced RAP since it reduces pro-inflammatory cytokine release rather than just inhibits cell death. However, mechanistic studies remain an unmet need, as we are not quite aware of feasible interventions that target the transcription and expression processes of PANoptosis-related genes in osteoblasts. In addition, more *in vivo* studies should be performed to determine whether inhibition of PANoptosis (or just apoptosis or pyroptosis or necroptosis) contributes to the reduction of osteoblast death and resolution of RAP.

#### *E. faecalis* and osteoblast differentiation

Bone remodelling is a sequential and orderly process that includes three stages: initiation, reversal, and termination. Osteoblasts play a key role in the reversal phase, where, in addition to inhibiting bone resorption, recruitment and differentiation of osteoblasts ultimately promote bone formation [111,112]. A study indicated that *E. faecalis* inhibits the osteogenic differentiation of ovine osteoblast-like cells [109]. HKEF downregulated the activity of the transcription factor Runx2 and the expression of osteogenic marker genes, which are typical features of osteoblast differentiation [108]. Further study demonstrated that the inhibitory effect of *E. faecalis* on osteogenic differentiation of pre-osteoblasts MC3T3-E1 is mainly dependent on the p38 pathway and partially on the ERK1/2 pathway [107]. These findings consistently revealed that *E. faecalis* infection inhibits osteoblast differentiation. Nevertheless, the understanding of the molecular mechanisms is insufficient, and more in-depth studies may contribute to exploring new therapeutic modalities by integrating the effects of *E. faecalis* on osteogenesis and the immune response.

### Implications of *E. faecalis*-modulated host cell responses for management of RAP

*E. faecalis* is a pathogen widely used in experimental infection models for research on the underlying pathogenesis of RAP. Interference with *E. faecalis*-modulated host cell responses may help to treat RAP. *In vivo* experiments demonstrated that inflammation and bone destruction were alleviated in RIPK3-deficient mice with *E. faecalis*-induced RAP [113]. Moreover, the CASP-1 inhibitor partly suppressed bone resorption in an experimental RAP rat model [114]. The challenge, however, is to which extent we should manipulate these pro-inflammatory pathways using inhibitors to prevent disease progression without compromising the further spread of infection. The molecular connections between different RCD modalities may help to switch a pro-inflammatory RCD mode (eg, pyroptosis and necroptosis) into a more immunologically silent one (eg, autophagy), thereby controlling infection with reduced inflammation. Hence, specific inhibitors targeting those molecular switches may be more potent for the management of RAP. Furthermore, autophagy is involved in the regulation of macrophage polarisation [115], suggesting that we can also consider the relationships between RCD and macrophage polarisation, rather than just the crosstalk between different RCD patterns. Indeed, RCT and microscopic apical surgery are the main clinical procedure for RAP. Another challenge is, at the different stages of RAP treatment, which agents targeting master regulators of *E. faecalis* or the host should be adopted as adjuncts and how to deliver these agents to the periapical zone with benefits to the patients. In future therapeutic strategies at the molecular level of RAP, further investigation of *E. faecalis*-modulated host cell responses and relevant signalling pathways will be of paramount importance, as it will lead to clinical translation to achieve the ideal effect.

## Discussion

More recently, *E. faecalis* has been widely recognised as the dominant species associated with RAP; however, the pathogenic mechanism of *E. faecalis* in RAP remains unclear. *E. faecalis* resists adverse conditions after RCT by altering the expression of relevant virulence genes and the synthesis and activity of biochemical components to maintain its biofilm formation capacity [116]. With advances in experimental techniques, mechanistic studies are no longer limited to the pathogenic properties of *E. faecalis*, but contributed to our understanding of host cell responses modulated by *E. faecalis*. To date, efforts have been made to elucidate the modulatory effects of *E. faecalis* on macrophages and osteoblasts. However, there is very little research on its regulatory effects and mechanisms on other immune cells, such as dendritic cells, lymphocytes, and neutrophils. As immune response is a complicated process that appears as a series of tissue reactions, different types of immune cells should be considered when exploring the pathogenic mechanisms of *E. faecalis* infection.

Previous studies have revealed that *E. faecalis* resists autophagy to promote intracellular survival in macrophages and inhibits macrophage apoptosis at low bacterial concentrations [59,60]. When bacteria proliferate to a certain concentration, *E. faecalis* induces macrophage PANoptosis and promotes an inflammatory response [58]. Additionally, *E. faecalis* may induce PANoptosis in osteoblasts [103,104]. Future research into the specific mechanisms involved in *E. faecalis*-modulated RCD in key cells should focus on the following: (i) Given the crucial role PANoptosis plays during infection, further investigation should shed some light on ways to modulate it for the promotion of pathogen removal or reduction of osteoblast death with minimised inflammatory pathology. Therefore, it is necessary to reveal specific mechanisms of PANoptosis activation in immune cells and osteoblasts, as well as the crosstalk with other pathways. (ii) Autophagy is a form of cellular self-degradation that rarely induces inflammation. It is awaited to investigate potential therapeutic targets to clear *E. faecalis* from the perspective of the molecular mechanisms of autophagy. (iii) Are there other types of RCD modulated by *E. faecalis* that play a dominant role?

Macrophages that remain viable after *E. faecalis* infection are polarised to an M2 or atypical M1 phenotype, causing persistent bacterial infection and chronic RAP [64,65]. M1 polarisation of macrophages is an essential protective mechanism against infection [91]. Hence, an improved understanding of the precise mechanisms and factors through which *E. faecalis* regulates macrophage polarisation will help to tune macrophage polarisation states, thereby removing pathogens early during infection.

The expression of virulence genes can lead to a spectrum of events favouring the development or maintenance of *E. faecalis* infection, as well as the progression of inflammation and tissue injury. However, it is not yet clear which virulence factors/genes are involved in the induction of host cell responses by *E. faecalis*. Conceivably, there are uncharacterised virulence factors/genes related to host-pathogen dynamics [117]. Prospective attempts should be conducted to decipher the complex genetic networks that regulate host-*E. faecalis* interactions.

Microbial factors, immune regulation, and bone homeostasis interact with each other and their complex associations make it challenging to precisely unravel the pathogenetic mechanisms involved in RAP [114,118–120]. The evolutionary interactions between *E. faecalis* and its host are never-ending. *E. faecalis* can develop new strategies to resist host defence mechanisms. A more in-depth study of the pathogenicity of microorganisms and the host responses they modulate is pivotal for detecting and completely removing pathogens from root canals, identifying potential therapeutic targets, controlling infection, and preventing the occurrence of RAP.
